# Supplementary material for: A Dyadic Perspective of Felt Security: Does Partners’ Security Buffer the Effects of Actors’ Insecurity on Daily Commitment?
Source: Int J Environ Res Public Health. 2020 Oct 12;17(20):7411. doi: 10.3390/ijerph17207411 (PMC7599705; doi:10.3390/ijerph17207411)
Supplement: Supplementary file 1 [file ijerph-17-07411-s001.pdf]

Online Supplemental Materials

These online supplemental materials include additional information on the sample and analyses presented in the paper, and outline additional analyses to support the interpretation of the results and conclusions presented in the paper.

Table of Contents

1. Further Information on Study Sample 1
1.1 Consideration of Power 1
1.2 Prior Use of Samples 1

2. RQ1: Dyadic Effects of Daily Felt Security on Commitment 2
2.1 SPSS Syntax 2
2.2 Between-Person Effects 2

3. RQ2: Dyadic Effects of Trait Insecurity on Commitment 4
3.1 SPSS Syntax 4

4. Integrated Data Analyses 5

5. Additional Analyses 6
5.1 Gender Differences 6
5.2 Differences across Relationship Length 7
5.3 Simultaneous Modeling of Actors' X Partners' Daily Felt Security and Attachment Anxiety 8
5.4 Testing Dyadic Effects of Attachment Avoidance on Commitment 9

1 Further Information on Study Sample

Dyadic studies are time and resource intensive, which has two important implications. First, sample sizes are necessarily constrained by funding and participant attrition, although the studies here were designed to overcome this limitation in prior research. Second, these studies are designed to examine multiple, independent processes. As outlined below, the aims, results and analyses presented in the current paper are independent of any reported in prior publications.

1.1 Consideration of Power

The sample sizes of Study 1 and 2 were determined based on prior research underpinning the multiple aims of broad projects examining actor and partner effects on daily dynamics. The target for each sample was 80 couples accounting for attrition due to non-compliance with the daily sampling procedure, which balanced funding with the aim to have adequate statistical power to detect small actor and partner effects based on prior studies and conventions at that time. However, the original aims of the study when funded did not specifically target actor X partner interactions. In addition, prior work on partner buffering effects indicating that partners might play a strong-link role have not examined these dyadic effects in daily interactions, and so we could not apply effect sizes from previous studies to consider a priori power analyses for these existing samples given the different measurement, context and analytic strategies across and within studies. Thus, we did not conduct a priori power analyses for the current investigation focused on actor X partner effects. In our Discussion section, we recognize the possibility that some effects may not have emerged due to limited power.

1.2 Prior Use of Samples

Prior papers using the data from Study 1 have focused on examining the degree to which individual factors entering the study, such as attachment insecurity and sexist attitudes, predict daily emotional and behavioral dynamics (Cross et al., 2019; Hammond & Overall, 2013; Overall et al., 2014, 2015, 2016), as well as the extent to which daily negative behaviors predict perceived partner responsiveness and relationship satisfaction (Overall, 2020; Sasaki & Overall, 2020). Study 2 was specifically designed to replicate the design and procedure of Study 1. As in Study 1, prior papers using the data from Study 2 have focused on examining the degree to which individual factors entering the study, such as attachment insecurity and sexist attitudes, predict daily emotional and behavioral dynamics (Cross, et al., 2017; Girme, Overall, Simpson & Fletcher, 2015; Overall et al., 2015), as well as the extent to which daily negative behaviors predict perceived partner responsiveness and relationship satisfaction (Overall, 2020; Sasaki & Overall, 2020). None of these prior studies have examined the degree to which actors' and partners' attachment insecurity and felt security are associated with commitment within daily life. Thus, all of the data and results presented in this paper are completely novel and independent of prior papers.

2 RQ1: Dyadic Effects of Daily Felt Security on Commitment

2.1 SPSS Syntax

We modelled the effects of within-person variations in (a) actors' felt security on day *i*, (b) partners' felt security on day *i*, and the (c) interaction between actors' and partners' felt security on day *i* predicting actors' commitment on day *i*, controlling for gender, the passage of time across the 21-day diary period, the corresponding between-person effects, and actors' commitment on day *i*-1 (Bolger & Laurenceau, 2013). The SPSS syntax is as follows:

```
MIXED CommD WITH gender day_c CommD_c_PD FeltSec_pc p_FeltSec_pc FeltSec_mean_c p_FeltSec_mean_c
  /FIXED= gender day_c CommD_c_PD FeltSec_pc p_FeltSec_pc FeltSec_pc*p_FeltSec_pc FeltSec_mean_c p_FeltSec_mean_c FeltSec_mean_c*p_FeltSec_mean_c
  /PRINT=SOLUTION TESTCOV COVB
  /RANDOM= woman man | SUBJECT(dyadid) COVTYPE(un)
  /REPEATED= obs | SUBJECT(dyadid*day) COVTYPE(CSH).
```

CommD = Commitment
CommD_c_PD = commitment on the previous day, grand-mean centered
Day_c = day of diary, centered so that zero is midway through the 21-day diary period
FeltSec_pc = actors' felt security, person-mean centered
p_FeltSec_pc = partners' felt security, person-mean centered
FeltSec_mean_c = average actors' felt security, grand-mean centered
p_FeltSec_mean_c = partners' felt security, grand-mean centered
dyadid = unique dyad identifier
obs = unique person identifier

2.2 Between-Person Effects

Table S1 presents the between-person effects in the same model that examined the within-person effects of actors' felt security, partners' felt security and actor X partner felt security on actors' daily commitment. Unlike the within-person components that represent daily variations in actors' or partners' felt security, between-person components reflect individual differences in average levels of actors' or partners' felt security across days. In both studies, higher average levels of actors' felt security across days was associated with higher daily commitment. In Study 1, but not Study 2, higher average levels of partners' felt security was also associated with higher commitment. Moreover, in both studies, the between-person effects of actor X partner felt security were significant. Decomposing the interaction revealed that higher average levels of actors' felt security across days was associated with higher daily commitment, but these increases were greater when partners also

reported higher average levels of felt security (Study 1: *b* = .60, *t* = 5.93, *p* < .001, *r* = .57; Study 2: *b* = .86, *t* = 10.20, *p* < .001, *r* = .78), compared to when partners reported lower average levels of felt security (Study 1: *b* = .33, *t* = 4.89, *p* < .001, *r* = .50; Study 2: *b* = .67, *t* = 11.01, *p* < .001, *r* = .80). Accordingly, the higher commitment experienced by actors higher in felt security (right side of figure) was enhanced when partners were also higher in felt security Study 1: *b* = .36, *t* = 3.91, *p* < .001, *r* = .42; Study 2: *b* = .19, *t* = 2.29, *p* = .025, *r* = .27). Hence, the between-person effects also support that partners' high felt security can have beneficial effects, including buffering days of low insecurity (as shown by the central within-person effects focused on in the paper) and bolstering the positive between-person effects of actors' security on commitment across days.

Table S1. The Between-Person Effects of Actor and Partner Daily Felt Security on Actors' Daily Commitment

	**Commitment**					
			95% CI			
Predictors	*B*	*t*	*Low*	*High*	*p*	*r*
**Study 1**						
Intercept	6.34	115.47	6.228	6.447	<.001	1.00
Actor Felt Security	.47	6.77	.330	.602	<.001	.50
Partner Felt Security	.22	3.37	.092	.352	.001	.30
Actor × Partner Felt Security	**.17**	**2.66**	**.043**	**.302**	**.010**	**.30**
**Study 2**						
Intercept	6.21	168.11	6.137	6.284	<.001	1.00
Actor Felt Security	.76	12.26	.641	.887	<.001	.73
Partner Felt Security	.09	1.50	-.029	.209	.137	.14
Actor × Partner Felt Security	**.14**	**2.47**	**.026**	**.247**	**.016**	**.29**

Note. The significant interaction effects presented in bold are presented in Figure S1. CI = confidence interval. Effect sizes (*r*) were computed using Rosenthal and Rosnow's (2007) formula: $r = \sqrt{(t2 / t2 + df)}$. In these multilevel models, the Satterthwaite approximation is applied to provide specific degrees of freedom for each effect, which were used to calculate the effect sizes.

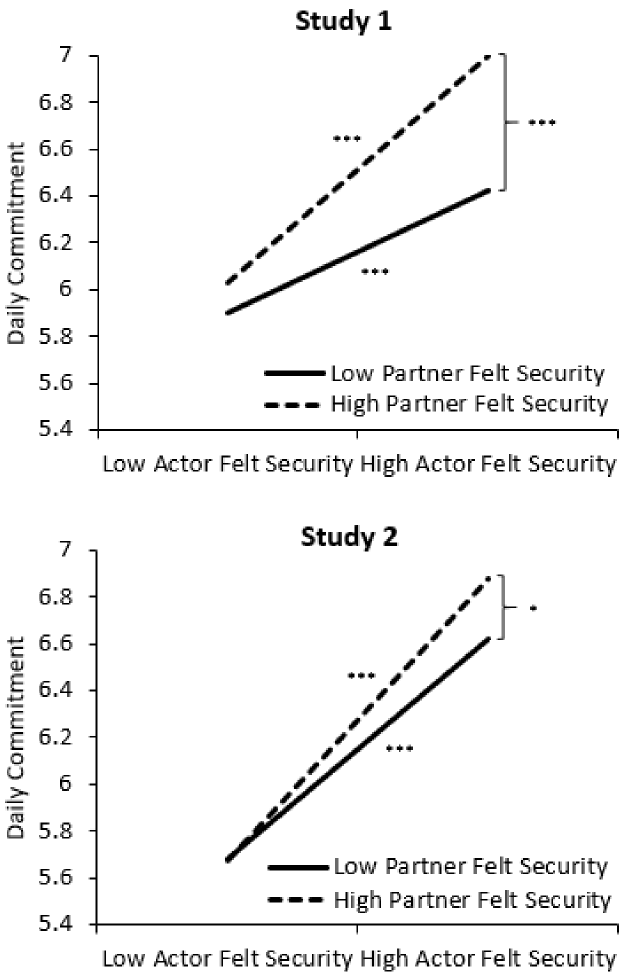

Figure S1. The between-person effects of Actor and Partner Felt Security on Daily Commitment in Studies 1 and 2.

Note. Low and high levels of Actor and Partner felt security represent 1 *SD* below and above the mean. The simple effects of the slopes and contrasts are marked $^{***}p < .001$ and $^{*}p < .05$.

3 RQ2: Dyadic Effects of Trait Insecurity on Commitment

3.1 SPSS Syntax

We modelled the effect of (a) actors' attachment anxiety, (b) partners' attachment anxiety, and the (c) interaction between actors' and partners' attachment anxiety predicting actors' daily commitment, controlling for gender, and the passage of time across the 21-day diary period (Kenny et al., 2006). The SPSS syntax is as follows:

MIXED CommD **WITH** gender day_c ATTANX_c p_ATTANX_c
/FIXED= gender day_c ATTANX_c p_ATTANX_c ATTANX_c*p_ATTANX_c
/PRINT=SOLUTION TESTCOV COVB
/RANDOM= woman man **| SUBJECT(**dyadid**) COVTYPE(**un**)**
/REPEATED= obs **| SUBJECT(**dyadid*day**) COVTYPE(**CSH**).**

CommD = Commitment

Day_c = day of diary, centered so that zero is midway through the 21-day diary period

ATTANX_c = actor attachment anxiety, grand-mean centered

p_ATTANX_c = partner attachment anxiety, grand-mean centered

dyadid = unique dyad identifier

obs = unique person identifier

4 Integrative Data Analyses

We conducted integrative data analyses by applying the same models examining RQ1 (as presented in Table 2) and RQ2 (as presented in Table 3) to the pooled data across both studies and included the main and interaction effects of study (-1 = Study 1, 1 = Study 2) to test whether the effects differed across studies.

RQ1. As shown in Table S2, study differences emerged in the main and interaction effects of actors' and partners' felt security on daily commitment. First, lower actors' felt security was associated with lower daily commitment, but this effect was stronger in Study 1 (b = .42, t = 22.49, p < .001, r = .32) than in Study 2 (b = .32, t = 16.55, p < .001, r = .24). Second, lower partners' felt security was associated with lower daily commitment in Study 2 (b = .10, t = 5.29, p < .001, r = .08), but not in Study 1 (b = .01, t = .48, p = .632, r = .01). Third, the effects of actor X partner felt security differed across studies (as shown in bold in Table S2). Consistent with what was reported in the paper, a strong-link pattern was found in both studies, but the strong-link pattern was stronger in Study 2 than in Study 1 (as shown in Figure 1).

Table S2. Integrated Data Analysis: The effects of Actor and Partner Daily Felt Security on Actors' Daily Commitment

	Commitment					
			95% CI			
	B	*t*	*Low*	*High*	*p*	*r*
Intercept	6.27	184.35	6.205	6.339	<.001	1.00
Study	-.08	-2.48	-.149	-.017	.014	.20
Actor Felt Security	.37	27.47	.341	.393	<.001	.38
Partner Felt Security	.06	4.14	.029	.083	<.001	.06
Actor × Partner Felt Security	-.08	-8.32	-.098	-.061	<.001	.17
Study × Actor Felt Security	-.05	-3.66	-.075	-.023	<.001	.05
Study × Partner Felt Security	.05	3.47	.020	.074	.001	.05
**Study × Actor × Partner Felt Security**	**-.02**	**-2.20**	**-.040**	**-.002**	**.028**	**.04**

Note. Analyses were conducted controlling for the corresponding between-person effects of felt security. CI = confidence interval. In these multilevel models, the Satterthwaite approximation is applied to provide specific degrees of freedom for each effect, which were used to calculate the effect sizes.

RQ2. As shown in Table S3, the effect of actor X partner attachment anxiety differed across studies (as shown in bold). Decomposing the interaction demonstrated a significant interaction revealing a strong-link pattern in Study 1 but not in Study 2 (similar to that shown in Figure 2).

Given that IDA revealed study differences in the effects of (1) actor X partner state felt security and (2) actor X partner trait insecurity, it is not appropriate to interpret the pooled effects from IDA. Instead, we present the effects for the two studies separately in the paper.

Table S3. Integrated Data Analysis: The effects of Actor and Partner Attachment Anxiety on Actors' Daily Commitment

	Commitment					
			95% CI			
Predictors	*B*	*t*	*Low*	*High*	*p*	*r*
Intercept	6.27	115.63	6.162	6.376	<.001	.99
Actor Attachment Anxiety	-.19	-4.36	-.280	-.106	<.001	.26
Partner Attachment Anxiety	-.16	-3.49	-.244	-.068	.001	.21
Actor × Partner Attachment Anxiety	-.05	-1.08	-.153	.045	.281	.09
Study	-.04	-.73	-.144	.066	.465	.06
Study × Actor Attachment Anxiety	.01	.19	-.077	.094	.848	.01
Study × Partner Attachment Anxiety	.10	2.40	.019	.191	.017	.15
**Study × Actor × Partner Attachment Anxiety**	**.15**	**2.99**	**.051**	**.249**	**.003**	**.24**

Note. CI = confidence interval. Effect sizes (*r*) were computed using Rosenthal and Rosnow's (2007) formula: $r = \sqrt{(t2 / t2 + df)}$. In these multilevel models, the Satterthwaite approximation is applied to provide specific degrees of freedom for each effect, which were used to calculate the effect sizes.

5 Additional Analyses

5.1 Gender Differences

To test whether gender moderated the effects of actors' and partners' felt security and attachment anxiety, we reran our analyses including all main and interaction effects with gender (-1 = women, 1 = men) separately for Studies 1 and 2.

Actor X Partner Felt Security. The effects were significant for both men and women, although there were some gender differences in the strength of the main effects. Gender differences

emerged for the main effect of actors' within-person variations in felt security on daily commitment in Studies 1 (*b* = -.07, *t* = -3.34, *p* = .001, *r* = .07) and 2 (*b* = -.06, *t* = -3.36, *p* = .001, *r* = .08). Days of lower actors' felt security predicted lower commitment, but this effect was stronger for women (*b* = .47, *t* = 18.03, *p* < .001, *r* = .35 in Study 1 and *b* = .38, *t* = 17.05, *p* < .001, *r* = .37 in Study 2) than men (*b* = .34, *t* = 11.55, *p* < .001, *r* = .23 in Study 1 and *b* = .25, *t* = 8.22, *p* < .001, *r* = .19 in Study 2). Tests of gender differences for the main effect of partners' felt security on daily commitment was not significant in Study 1 (*b* = -.01, *t* = -.42, *p* = .672, *r* = .01), but was significant in Study 2 (*b* = .04, *t* = 2.00, *p* = .046, *r* = .05). In Study 2, days of lower partners' felt security predicted lower commitment, but this effect was stronger for men (*b* = .15, *t* = 4.69, *p* < .001, *r* = .11) than women (*b* = .07, *t* = 3.23, *p* = .001, *r* = .08). Most relevant to the current aims, despite gender differences in some of the main effects, there was no gender differences in the interaction effects of actors' X partners' felt security on daily commitment in Study 1 (*b* = .01, *t* = .59, *p* = .552, *r* = .02) or Study 2 (*b* = -.02, *t* = -1.34, *p* = .179, *r* = .04).

Actor X Partner Attachment Anxiety. One marginally significant gender difference emerged for the main effect of actors' attachment anxiety on daily commitment in Study 1 (*b* = -.11, *t* = -1.86, *p* = .066, *r* = .16) but not Study 2 (*b* = .03, *t* = .35, *p* = .730, *r* = .04). In Study 1, men higher in attachment anxiety reported lower daily commitment (*b* = -.32, *t* = -3.29, *p* = .001, *r* = .28), but this was not found for women (*b* = -.10, *t* = -1.41, *p* = .163, *r* = .12). More relevant to the current aims, there was no gender differences in the main effect of partners' attachment anxiety (Study 1: *b* = -.07, *t* = -1.17, *p* = .246, *r* = .10; Study 2: *b* = .02, *t* = .29, *p* = .770, *r* = .03) or the actors' X partners' attachment anxiety interaction (Study 1: *b* = -.07, *t* = -1.45, *p* = .151, *r* = .17; Study 2: *b* = -.03, *t* = -.45, *p* = .652, *r* = .05).

5.2 Differences across Relationship Length

We first tested whether relationship length was associated with daily felt security, attachment anxiety, and daily commitment. Only one significant effect emerged: longer relationship length was associated with lower attachment anxiety in Study 1 (*b* = -.01, *t* = -2.17, *p* = .033, *r* = .24). No other associations between relationship length, felt security and commitment emerged.

Next, to test whether relationship length moderated the effects of actors' and partners' felt security and attachment anxiety, we reran our analyses including all main and interaction effects with relationship length separately for Studies 1 and 2.

Actor X Partner Felt Security. Differences across relationship length emerged for the main effect of actors' within-person variations in felt security on daily commitment in Studies 1 (*b* = -.00, *t* = -2.50, *p* = .012, *r* = .05) and 2 (*b* = -.00, *t* = -3.32, *p* = .001, *r* = .07). Days of lower actors' felt security predicted lower commitment, but this effect was stronger in shorter relationships (*b* = .47, *t* = 17.22, *p* < .001, *r* = .34 in Study 1 and *b* = .41, *t* = 14.78, *p* < .001, *r* = .31 in Study 2) than in longer relationships (*b* = .36, *t* = 11.19, *p* < .001, *r* = .23 in Study 1 and *b* = .26, *t* = 9.11, *p* < .001, *r* = .20 in

Study 2). There were no differences across relationship length in the main effect of partners' felt security (Study 1: *b* = .00, *t* = 1.09, *p* = .277, *r* = .02; Study 2: *b* = -.00, *t* = -1.19, *p* = .233, *r* = .03) or the actors' X partners' felt security interaction (Study 1: *b* = -.00, *t* = -.33, *p* = .744, *r* = .01; Study 2: *b* = .00, *t* = -.67, *p* = .500, *r* = .02).

Actor X Partner Attachment Anxiety. No differences across relationship length emerged in the main effect of actors' attachment anxiety (Study 1: *b* = -.01, *t* = -1.64, *p* = .105, *r* = .16; Study 2: *b* = -.00, *t* = -1.21, *p* = .230, *r* = .11), partners' attachment anxiety (Study 1: *b* = -.00, *t* = -1.07, *p* = .289, *r* = .11; Study 2: *b* = -.00, *t* = -.16, *p* = .875, *r* = .01) or the actors' X partners' attachment anxiety interaction (Study 1: *b* = -.00, *t* = -.76, *p* = .449, *r* = .09; Study 2: *b* = .00, *t* = .27, *p* = .790, *r* = .03).

5.3 Simultaneous Modelling of Actors' and Partners' Daily Felt Security and Attachment Anxiety

Simultaneously modelling the main and interaction effects of both (1) actors' and partners' daily felt security and (2) actors' and partners attachment anxiety on daily commitment produced identical results as when modelling felt security and attachment anxiety separately (see Table S4).

Table S4. The effects of Actor and Partner Daily Felt Security and Actor and Partner Attachment Anxiety on Actors' Daily Commitment

	**Commitment**					
			95% CI			
Predictors	*B*	*t*	*Low*	*High*	*p*	*r*
**Study 1**						
Intercept	6.34	117.76	6.232	6.446	<.001	1.00
Actor Felt Security	.42	21.50	.382	.458	<.001	.41
Partner Felt Security	.01	.56	-.027	.049	.577	.01
Actor × Partner Felt Security	**-.07**	**-4.22**	**-.102**	**-.037**	**<.001**	**.12**
Actor Attachment Anxiety	.03	.64	-.067	.132	.523	.06
Partner Attachment Anxiety	-.09	-1.70	-.191	.014	.091	.15
Actor × Partner Attachment Anxiety	**-.11**	**-2.16**	**-.212**	**-.008**	**.034**	**.25**
**Study 2**						
Intercept	6.21	162.73	6.130	6.283	<.001	1.00
Actor Felt Security	.34	18.51	.306	.378	<.001	.38
Partner Felt Security	.10	5.31	.061	.133	<.001	.12
Actor × Partner Felt Security	**-.10**	**-5.38**	**-.142**	**-.066**	**<.001**	**.16**
Actor Attachment Anxiety	-.04	-1.19	-.114	.029	.238	.11
Partner Attachment Anxiety	.04	1.11	-.031	.111	.272	.10
Actor × Partner Attachment Anxiety	-.01	-.16	-.067	.057	.873	.02

Note. Analyses were conducted controlling for the corresponding between-person effects of felt security. CI = confidence interval. Effect sizes (*r*) were computed using Rosenthal and Rosnow's (2007) formula: $r = \sqrt{(t\ 2 / t\ 2 + df)}$. In these multilevel models, the Satterthwaite approximation is applied to provide specific degrees of freedom for each effect, which were used to calculate the effect sizes.

5.4 Testing Dyadic Effects of Attachment Avoidance on Commitment

Our paper focuses on attachment anxiety because it captures the tendency to appraise, experience and monitor felt security (Fraley & Shaver, 1998, 2000) that is key to our theoretical framework. In contrast, attachment avoidance reflects a motivational orientation that continually down-regulates closeness and dependence (rather than reflexively monitors felt-security to obtain closeness and dependence). While it is not part of the core aims of the current paper, the tendency for avoidant actors and partners to deal with insecurity by distancing from the other should also interfere with commitment (Mikulincer & Shaver, 2007; Pistole et al., 1995). Thus, we reran the analyses modelling the main and interaction effects of actors' and partners' attachment avoidance on daily commitment.

As shown in Table S5, across Studies 1 and 2, a main effect of actors' attachment avoidance on daily commitment emerged such that high actors' attachment avoidance was associated with lower daily commitment, but the actor X partner attachment avoidance was not significant. Simultaneously modelling the main and interaction effects of both (a) actors' and partners' attachment avoidance and (b) actors' and partners' attachment anxiety on daily commitment did not alter the effects of attachment anxiety (see Table S6).

Table S5. The effects of Actor and Partner Attachment Avoidance on Actors' Daily Commitment

	Commitment					
			95% CI			
Predictors	*B*	*t*	*Low*	*High*	*p*	*r*
**Study 1**						
Intercept	6.36	81.85	6.204	6.513	<.001	.99
Actor Attachment Avoidance	-.22	-3.84	-.328	-.105	<.001	.34
Partner Attachment Avoidance	.02	.26	-.100	.131	.792	.02
Actor × Partner Attachment Avoidance	-.05	-.66	-.200	.100	.509	.08
**Study 2**						
Intercept	6.22	73.38	6.050	6.388	<.001	.99
Actor Attachment Avoidance	-.15	-1.97	-.309	.001	.052	.18
Partner Attachment Avoidance	-.06	-.75	-.215	.097	.457	.07
Actor × Partner Attachment Avoidance	.09	.85	-.121	.299	.401	.10

Note. CI = confidence interval. Effect sizes (*r*) were computed using Rosenthal and Rosnow's (2007) formula: $r = \sqrt{(t\ 2 / t\ 2 + df)}$. In these multilevel models, the Satterthwaite approximation is applied to provide specific degrees of freedom for each effect, which were used to calculate the effect sizes.

Table S6. The effects of Actor and Partner Attachment Anxiety and Actor and Partner Attachment Avoidance on Actors' Daily Commitment

	**Commitment**					
			95% CI			
Predictors	*B*	*t*	*Low*	*High*	*p*	*r*
**Study 1**						
Intercept	6.35	94.66	6.217	6.485	<.001	1.00
Actor Attachment Anxiety	-.20	-3.30	-.315	-.079	.001	.29
Partner Attachment Anxiety	-.25	-4.10	-.366	-.127	<.001	.35
Actor × Partner Attachment Anxiety	-.21	-3.03	-.349	-.072	.003	.34
Actor Attachment Avoidance	-.19	-3.44	-.303	-.082	.001	.30
Partner Attachment Avoidance	.06	.98	-.057	.169	.329	.08
Actor × Partner Attachment Avoidance	-.12	-1.73	-.260	.018	.087	.20
**Study 2**						
Intercept	6.21	73.50	6.039	6.377	<.001	.99
Actor Attachment Anxiety	-.17	-2.41	-.302	-.030	.017	.21
Partner Attachment Anxiety	-.02	-.22	-.153	.122	.824	.02
Actor × Partner Attachment Anxiety	.08	1.06	-.071	.232	.291	.13
Actor Attachment Avoidance	-.10	-1.29	-.260	.055	.201	.12
Partner Attachment Avoidance	-.04	-.48	-.199	.122	.635	.04
Actor × Partner Attachment Avoidance	.04	.33	-.181	.253	.740	.04

Note. CI = confidence interval. Effect sizes (*r*) were computed using Rosenthal and Rosnow's (2007) formula: $r = \sqrt{(t\,2 / t\,2 + df)}$. In these multilevel models, the Satterthwaite approximation is applied to provide specific degrees of freedom for each effect, which were used to calculate the effect sizes.

References

Bolger, N., & Laurenceau, J-P. (2013). *Intensive longitudinal methods: An introduction to diary and experience sampling research*. Guilford.

Cross, E. J., Overall, N. C., Hammond, M. D., & Fletcher, G. J. O. (2017). When Does Men's Hostile Sexism Predict Relationship Aggression? The Moderating Role of Partner Commitment. *Social Psychological and Personality Science*, *8*(3), 331–340. https://doi.org/10.1177/1948550616672000

Cross, E.J., Overall, N.C., Low, R.S.T., & McNulty, J.K. (2019). Men's hostile sexism, biased perceptions of low power, and aggression toward intimate partners. *Journal of Personality and Social Psychology*, *117*(2), 338-363. https://doi.org/10.1037/pspi0000167

Fraley, R. C., & Shaver, P. R. (1998). Airport separations: A naturalistic study of adult attachment dynamics in separating couples. *Journal of Personality and Social Psychology*, *75*(5), 1198–1212. https://doi.org/10.1037/0022-3514.75.5.1198

Fraley, R. C., & Shaver, P. R. (2000). Adult romantic attachment: Theoretical developments, emerging controversies, and unanswered questions. *Review of General Psychology*, *4*(2), 132–154. https://doi.org/10.1037/1089-2680.4.2.132

Girme, Y. U., Overall, N. C., Simpson, J. A., & Fletcher, G. J. O. (2015). "All or nothing": Attachment avoidance and the curvilinear effects of partner support. *Journal of Personality and Social Psychology*, *108*(3), 450–475. https://doi.org/10.1037/a0038866

Hammond, M. D., & Overall, N. C. (2013). Men's Hostile Sexism and Biased Perceptions of Intimate Partners: Fostering Dissatisfaction and Negative Behavior in Close Relationships. *Personality and Social Psychology Bulletin*, *39*(12), 1585–1599. https://doi.org/10.1177/0146167213499026

Kenny, D. A., Kashy, D. A., & Cook, W. L. (2006). *Dyadic data analysis*. Guilford.

Mikulincer, M., & Shaver, P. R. (2007). *Attachment in adulthood: Structure, dynamics, and change*. Guilford.

Overall, N. C. (2020). Behavioral variability reduces the harmful longitudinal effects of partners' negative-direct behavior on relationship problems. *Journal of Personality and Social Psychology*. Advance online publication. https://doi.org/10.1037/pspi0000231

Overall, N. C., Fletcher, G. J. O., Simpson, J. A., & Fillo, J. (2015). Attachment insecurity, biased perceptions of romantic partners' negative emotions, and hostile relationship behavior. *Journal of Personality and Social Psychology*, *108*(5), 730–749. https://doi.org/10.1037/a0038987

Overall, N. C., Girme, Y. U., Lemay, E. P., & Hammond, M. D. (2014). Attachment anxiety and reactions to relationship threat: The benefits and costs of inducing guilt in romantic partners. *Journal of Personality and Social Psychology*, *106*(2), 235–256. https://doi.org/10.1037/a0034371

Overall, N. C., Hammond, M. D., McNulty, J. K., & Finkel, E. J. (2016). When power shapes interpersonal behavior: Low relationship power predicts men's aggressive responses to low situational power. *Journal of Personality and Social Psychology*, *111*(2), 195–217. https://doi.org/10.1037/pspi0000059

Pistole, M. C., Clark, E. M., & Tubbs, A. L. (1995). Love relationships: Attachment style and the investment model. *Journal of Mental Health Counseling*, *17*(2), 199–209.

Sasaki, E., & Overall, N.C (2020). Partners' withdrawal when actors behave destructively: Implications of partners' responsiveness and relationship satisfaction. *Personality and Social Psychology Bulletin*. Advance online publication. https://doi.org/10.1177/0146167220926820

Rosenthal, R., & Rosnow, R. L. (2007). *Essentials of behavioral research: Methods and data analysis* (3rd ed.). McGraw-Hill.
